# Editorial: Bioactive compounds present in essential oils: Advances and pharmacological applications

**DOI:** 10.3389/fphar.2023.1130097

**Published:** 2023-01-11

**Authors:** Mozaniel Santana de Oliveira, Oliviu Vostinaru, Daniela Rigano, Eloisa Helena de Aguiar Andrade

**Affiliations:** ^1^ Museu Paraense Emílio Goeldi, Belém, Brazil; ^2^ Iuliu Hațieganu University of Medicine and Pharmacy, Cluj-Napoca, Romania; ^3^ Department of Pharmacy, University of Naples Federico II, Naples, Italy

**Keywords:** natural products, essential oils, medicinal plants (herbal drugs), traditional medicine, extractions

Compounds of natural origin have been used for centuries in traditional medicine, whether as extracts, resins, or essential oils. Essential oils, in particular, are extracted from aromatic plants, being the subject of study in almost all countries. Their complex mixture of volatile substances has delayed the potential use of biological activities that may be of industrial interest. Essential oils are volatile substances formed by the secondary metabolism of aromatic plants; their constituents usually have low molecular weight. However, some natural factors, such as physiological variations, environmental conditions, geographic variations, genetic factors, and plant evolution can alter these oils’ chemical composition and yield. Essential oils are widely studied in the pharmaceutical industry, mainly for their potential applications as biologically active agents. Over the years, several pharmacological activities have been demonstrated for the volatile compounds, such as antioxidant, anticancer, antiprotozoal, antimicrobial, anti-inflammatory, phytotoxic, and, neuroprotective (Rout et al., 2022).

Essential oils show a wide variety of constituents, mainly monoterpenes, sesquiterpenes, benzenoids, and phenylpropanoids. Species of the Amaranthaceae family, for example, are rich in bioactive compounds that are beneficial to human health, the main ones being α-terpinene, δ-3-carene, limonene, thymol, carvacrol, γ-terpinene, α-terpinolene, piperitone oxide, geraniol, α-pinene, β-pinene, iso-ascaridole, β-myrcene, α-ocimene, β-ocimene, citronellyl acetate, β-phellandrene, dihydroascaridole, trans-pinocarveol, carvone, piperitone, p-cymene, 4-carene, δ-3-carene, fenchone, linalool, menthone, nerol, β-pinene, pulegone, terpineol-4-ol, thujone, and iso-ascaridole. In addition, these compounds were associated with various biological activities like antibacterial, antiviral, anti-leishmanial, antioxidant, and anticancer effects (Dagni et al.).

Another important family for producing essential oils is the Apiaceae, particularly the species *Deverra tortuosa* DC. and *Deverra triradiata* Hochst. (Kamel et al.). for example, they used different extraction techniques to evaluate the yield and chemical composition, such as hydrodistillation, in addition, to two unconventional ones such as Microwave Assisted Hydrodistillation and Supercritical CO_2_ Extraction: evaluating fresh and dry samples. The results showed that the yield of essential oils (EOs) can be directly affected when preparing the sample, and the EOs extraction process. In addition, the results show that an unconventional extraction technique such as supercritical CO2, can be a viable alternative for the advancement in the processing of essential oils, as this extraction technique is also considered selective and green depending on the operational parameters. The results showed that the chemical composition was also qualitatively and quantitatively affected. There is a noticeable difference in the *D. tortuosa* oil, where the percentages of oxygenated compounds are 23.52, 23.03, and 49.74 for hydrodistillation (HD), microwave-assisted hydrodistillation (MADH), and supercritical fluids extraction (SFE), respectively, and the percentage of non-oxygenated compounds was 74.54 (HD), 73.02 (MADH), and 41.19 (SFE). These species are rich in terpenoids and phenylpropanoids such as sabinene, terpinen-4-ol, β-myrcene, α-terpinene, γ-terpinene, β-myrcene, germacrene D, myristicin, elemicin, and β-eudesmol. The results of biological activity demonstrate that with this study new formulations such as nanoemulsion were made from essential oils of *Deverra* species to treat and heal wounds. The formulation also exhibited anti-inflammatory and antioxidant activities, growth factor, of the hydroxyproline levels, and demonstrated complete re-epithelialization associated with activated hair follicles and abundant collagen fibres.

It is essential to highlight that when tested individually as isolated substances, volatile compounds also exhibited promising biological activities, being considered research targets for developing new drugs. Thymol is an example of a compound that affects the excitation and contraction of skeletal and smooth muscle. In addition, at higher concentrations, thymol can completely suppress acetylcholine-induced contractions in both the ileum and uterus, suggesting that both organs may respond to thymol treatment. Thus, thymol, as one of the chemical constituents of *Thymus vulgaris* L. essential oil, may explain the pharmacological effects used in traditional medicine to treat menstrual cramps (Premrov Bajuk et al.).

Plants from the Caprifoliaceae family, often used in traditional medicine, constitute another example of advances in studies related to the biological activity of volatile compounds. Pre-clinical studies suggested that several species like *Valeriana* sp or *Lonicera japonica* Thunb. Have essential pharmacological properties. They are sedative, hypnotic, antispasmodic, analgesic, antidepressant, anxiolytic, anticonvulsant, neuroprotective, antibacterial, antiviral, cytotoxic, but also anti-inflammatory, antioxidant, hepatoprotective, anti-gallstones, hypotensive, hypolipidemic, anti-thrombotic, antiallergic, and immunoregulatory (Li et al.; Zheng et al.). The importance of these studies, among others, is that they may support the development of herbal medicines. In this Research Topic, five articles were published, all bringing significant advances on compounds of natural origin. Research on natural products can be the key to the discovery of new chemically and biologically active substances, capable of a wide range of pharmacological applications presented in this topic, from an antibacterial effect with the reduction of bacterial resistance to anti-inflammatory activity, wound healing properties or the reduction of colic, among others.
